# Supplementary material for: Emergent invariance and scaling properties in the collective return dynamics of a stock market
Source: PLoS One. 2024 Feb 23;19(2):e0298789. doi: 10.1371/journal.pone.0298789 (PMC10889862; doi:10.1371/journal.pone.0298789)
Supplement: S1 File — Reporting the Complementary Cumulative Distribution Functions (CCDF) of returns, scaling and function invariance for a 3-month investment horizon, an interpretation of the Stretched Exponential model, and a derivation of the Vicsek model update rule. (PDF) [file pone.0298789.s001.pdf]

Supporting Information

S1 Appendix. Complementary Cumulative Distribution Functions (CCDF) of the Returns as a function of τ and year

In Fig. 1, we demonstrate that the CCDF of the returns follows a power law (scaling regime highlighted in cyan) similar to those observed in prior work (see Sec. Our Contributions).

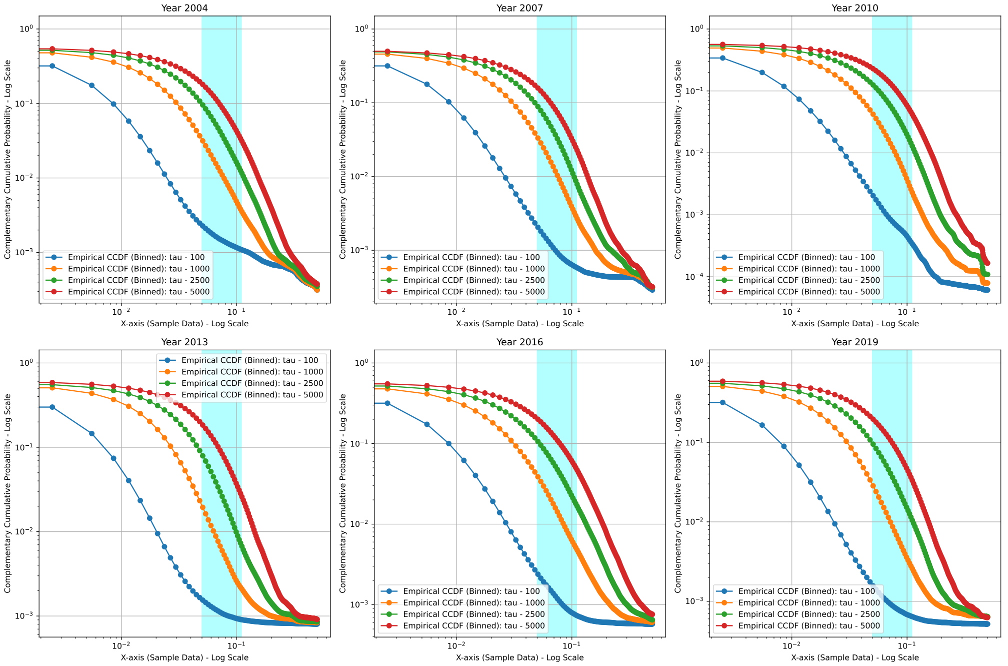

Fig 1. Empirically estimated CCDFs of returns (+ve values only): The CCDF of returns computed for different return horizons τ and years are presented. In accordance to prior work, power laws are observed for a finite range of τ (marked in cyan).

S2 Appendix. The expected value (mean) of the partial correlations $\mathbb{E}(c_{ij})$ does not scale as a function of τ

In Fig. 2, we demonstrate that the mean value of the partial correlations – as opposed to the standard deviation – of the returns does not scale as a function of τ.

S3 Appendix. Interpretability and emergent properties of the Stretched Exponential fit

We observed that in addition to the Power Law fit, the Stretched Exponential also explains the dependence of $b(\tau)$ with respect to τ. There is one extra model parameter (3 vs. 2). Similar to the effect of λ in the case of the Power Law, in this section, we evaluate whether the stretching parameter β in the Stretched Exponential fit can act as a market indicator.

To get a better sense of the behavior of the stretched exponential, in Fig. 3, we plot the $b(\tau) - \tau$ dependence in linear-log scale across 4 years: $2004, 2008, 2012, 2020$. Both an exponential fit – $\beta = 1$, linear in the linear-log scale – and the best stretched-exponential ($\beta < 1$) fits are plotted. As corroborated by the MAS experiments (see Fig.7), the stretched exponential is a significantly better fit.

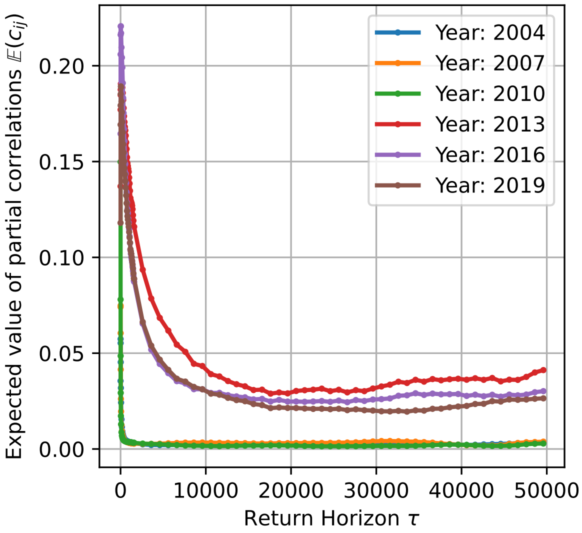

Fig 2. Expected value of the partial correlations as a function of τ and across years: The mean value of c_{ij} as a function of τ is presented. While the standard deviation shows scaling properties, we do not observe a similar relationship governing the means.

β appears to be a parameter that changes year-to-year. For year 2020, the exponential and stretched exponential fits are more similar to one another ($\beta \to 1$) than in 2004. This motivates investigating whether there is a general trend in the β values, similar to the λ's presented in the main paper. In Fig. 4, we show a similar plot.

First we explain the increasing trend: As β increases (tends to 1), the corresponding Stretched Exponential model fit flattens such that the short-term (small τ) and long-term (large τ) investment horizons are similarly effective. This can be confirmed by the y-intercepts in Fig. 3.

Note that this interpretation is similar to when λ is small in the Power Law fit. In fact, there are also corresponding anomalies (marked in cyan) for the years when the market was volatile – $2008, 2020$. Observe that for these years, the β values significantly overshoot the linear trend (beyond an error margin; 4-fold cross validation).

S4 Appendix. Experiments with reduced $T_{int} = 3$ months (Q2: Apr. 1 to June 30)

In this section, we demonstrate that (a) the functional form of the standardized PDFs $\hat{p}_\tau(x)$ is stable for some $\tau > \tau_0$ when $T_{int} = 3$ months between Apr. 1 and June 30 (Q2), and (b) the scaling phenomenon is once again observed between $b(\tau)$ and τ. We accordingly decrease the maximum investment horizon from 50000 minutes to 10000 minutes since $\tau < T_{int}$. Below we provide 3 figures corresponding to (a) the functional invariance (Fig. 5), (b) the scaling phenomenon (Fig. 6), and (c) model architecture search (Fig. 7). Note their similarity to those presented in the main text.

S5 Appendix. Deriving the Modified Vicsek model

We first review the Vicsek model. Let us consider a two-dimensional system of N kinetic particles. We denote, by $\vec{x}_i(t)$ and $\vec{v}_i(t)$, the position and velocity of particle i at time t. The position of i is updated by

$$\vec{x}_i(t + \Delta t) = \vec{x}_i(t) + \vec{v}_i(t)\Delta t. \tag{1}$$

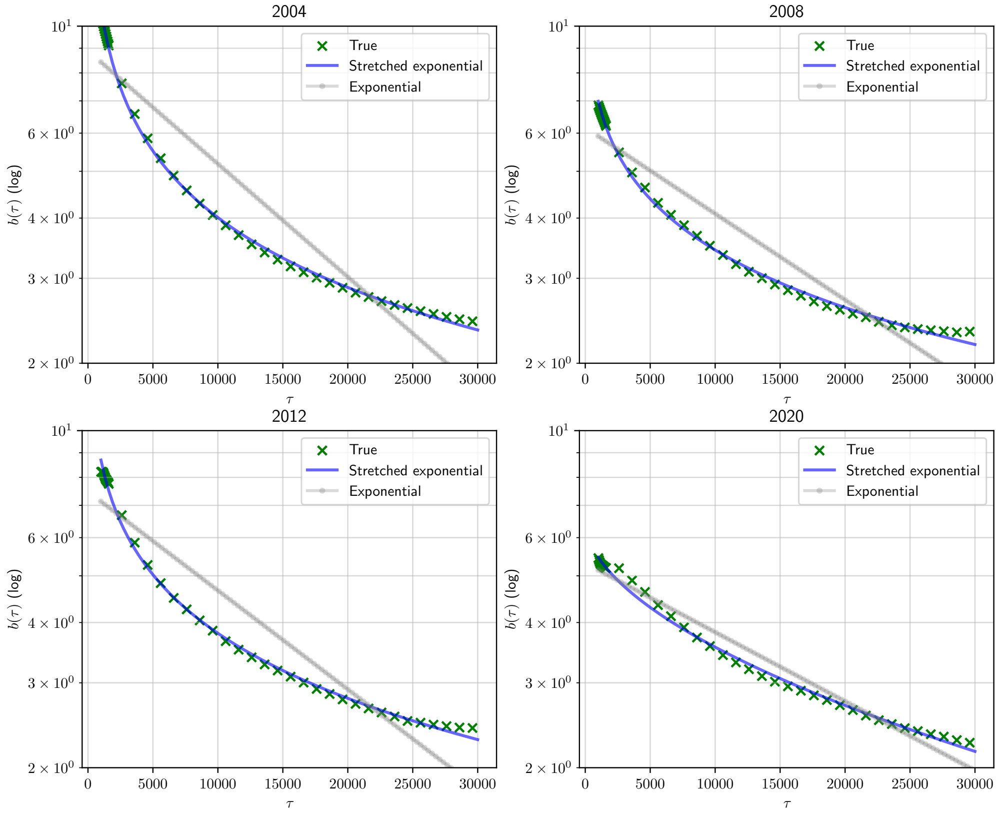

Fig 3. Plotting the dependence of $b(\tau)$ with respect to τ as a stretched exponential: Four log-linear plots (-X-) representing the relationship between $b(\tau)$ and τ are presented for years $2004, 2008, 2012, 2020$. The stretched exponential (-, green) and exponential (grey) fits are shown. Notice that for later years, the stretched exponential fit and the exponential fit are closer to each other.

The amplitude of the velocity of each particle is constant: $\|\vec{v}_i(t)\|_{\mathrm{F}} = v_{\mathrm{abs}}$. But the direction of $\vec{v}_i(t)$ is update by

$$\vec{v}_i(t) = v_{\mathrm{abs}} \begin{bmatrix} \cos\theta_i(t) \\ \sin\theta_i(t) \end{bmatrix}, \tag{2}$$

and

$$\theta_i(t + \Delta t) = \frac{1}{N_{i,\delta}} \sum_{j:\|\vec{x}_i(t) - \vec{x}_j(t)\|_{\mathrm{F}} < \delta} \theta_j(t) + \Xi_i(t),$$
$$\Xi_i(t) \sim [-\eta/2, \eta/2], \tag{3}$$

where $N_{i,\delta}$ is the number of elements j that satisfy $\|\vec{x}_i(t) - \vec{x}_j(t)\|_{\mathrm{F}} < \delta$. Instead of the uniform distribution, we can use the normal distribution for $\Xi_i(t)$:

$$\Xi_i(t) \sim \mathcal{N}\left(0, \frac{\eta^2}{12}\right). \tag{4}$$

Continuous version of the Vicsek model: The continuous variant of Eqs. (1), (2), and (3), takes the form

$$\frac{d}{dt}\vec{x}_i(t) = \vec{v}_i(t), \tag{5}$$

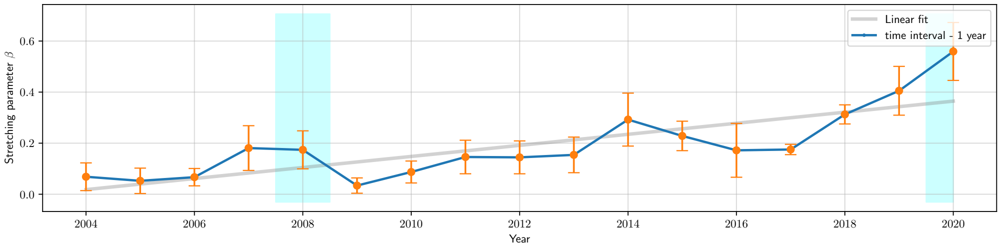

Fig 4. Stretching parameter β with respect to year: Values of β from 2004 to 2020 with interval $T_{int} = 1$ year are plotted above along with a linear trendline fit. Error bars are generated using 4-fold cross-validation. Years marked with anomalies are identified in the cyan-shaded regions.

where

$$\vec{v}_i(t) = v_{\text{abs}} \begin{bmatrix} \cos\theta_i(t) \\ \sin\theta_i(t) \end{bmatrix}, \tag{6}$$

$$\dot{\theta}_i(t) = -\alpha_i\theta_i(t) + \frac{\beta_i}{N_{i,\delta}} \sum_{j:\|\vec{x}_i(t)-\vec{x}_j(t)\|_{\text{F}}<\delta} \theta_j(t) + \xi_i(t). \tag{7}$$

Taking the time-derivative of Eq. (5), we can transform it into

$$\dot{\vec{v}}_i(t) = v_{\text{abs}} \begin{bmatrix} -\sin\theta_i(t) \\ \cos\theta_i(t) \end{bmatrix} \dot{\theta}_i(t) \tag{8}$$

$$= v_{\text{abs}} \begin{bmatrix} -\sin\theta_i(t) \\ \cos\theta_i(t) \end{bmatrix} \Bigg(-\alpha_i\theta_i(t) + \frac{\beta_i}{N_{i,\delta}} \sum_{j:\|\vec{x}_i(t)-\vec{x}_j(t)\|_{\text{F}}<\delta} \theta_j(t) + \xi_i(t) \Bigg). \tag{9}$$

Again, we discretize the above equations, Eqs. (5), (6), and (6):

$$\vec{x}_i(t+\Delta t) = \vec{x}_i(t) + \vec{v}_i(t)\Delta t, \tag{10}$$

where

$$\vec{v}_i(t) = v_{\text{abs}} \begin{bmatrix} \cos\theta_i(t) \\ \sin\theta_i(t) \end{bmatrix}, \tag{11}$$

and

$$\theta_i(t+\Delta t) = \theta_i(t) + \Bigg(-\alpha_i\theta_i(t) + \frac{\beta_i}{N_{i,\delta}} \sum_{j:\|\vec{x}_i(t)-\vec{x}_j(t)\|_{\text{F}}<\delta} \theta_j(t) + \xi_i(t) \Bigg)\Delta t$$

$$= (1-\alpha_i\Delta t)\theta_i(t) + \frac{\beta_i\Delta t}{N_{i,\delta}} \sum_{j:\|\vec{x}_i(t)-\vec{x}_j(t)\|_{\text{F}}<\delta} \theta_j(t) + \xi_i(t)\Delta t. \tag{12}$$

To recover the original equation of $\theta_i(t)$, Eq. (3), we need to set $\alpha_i = \frac{1}{\Delta t}$ and $\beta_i = \frac{1}{\Delta t}$. To summarize the above equations, Eqs. (10), (11), and (12), we get

$$\vec{v}_i(t+\Delta t) = \vec{v}_i(t) + v_{\text{abs}} \begin{bmatrix} -\sin\theta_i(t) \\ \cos\theta_i(t) \end{bmatrix} \Bigg(-\alpha_i\theta_i(t) + \frac{\beta_i}{N_{i,\delta}} \sum_{j:\|\vec{x}_i(t)-\vec{x}_j(t)\|_{\text{F}}<\delta} \theta_j(t) + \xi_i(t) \Bigg)\Delta t$$

$$= v_{\text{abs}} \begin{bmatrix} \cos\theta_i(t) \\ \sin\theta_i(t) \end{bmatrix} + v_{\text{abs}} \begin{bmatrix} -\sin\theta_i(t) \\ \cos\theta_i(t) \end{bmatrix} \Bigg(-\alpha_i\theta_i(t) + \frac{\beta_i}{N_{i,\delta}} \sum_{j:\|\vec{x}_i(t)-\vec{x}_j(t)\|_{\text{F}}<\delta} \theta_j(t) + \xi_i(t) \Bigg)\Delta t. \tag{13}$$

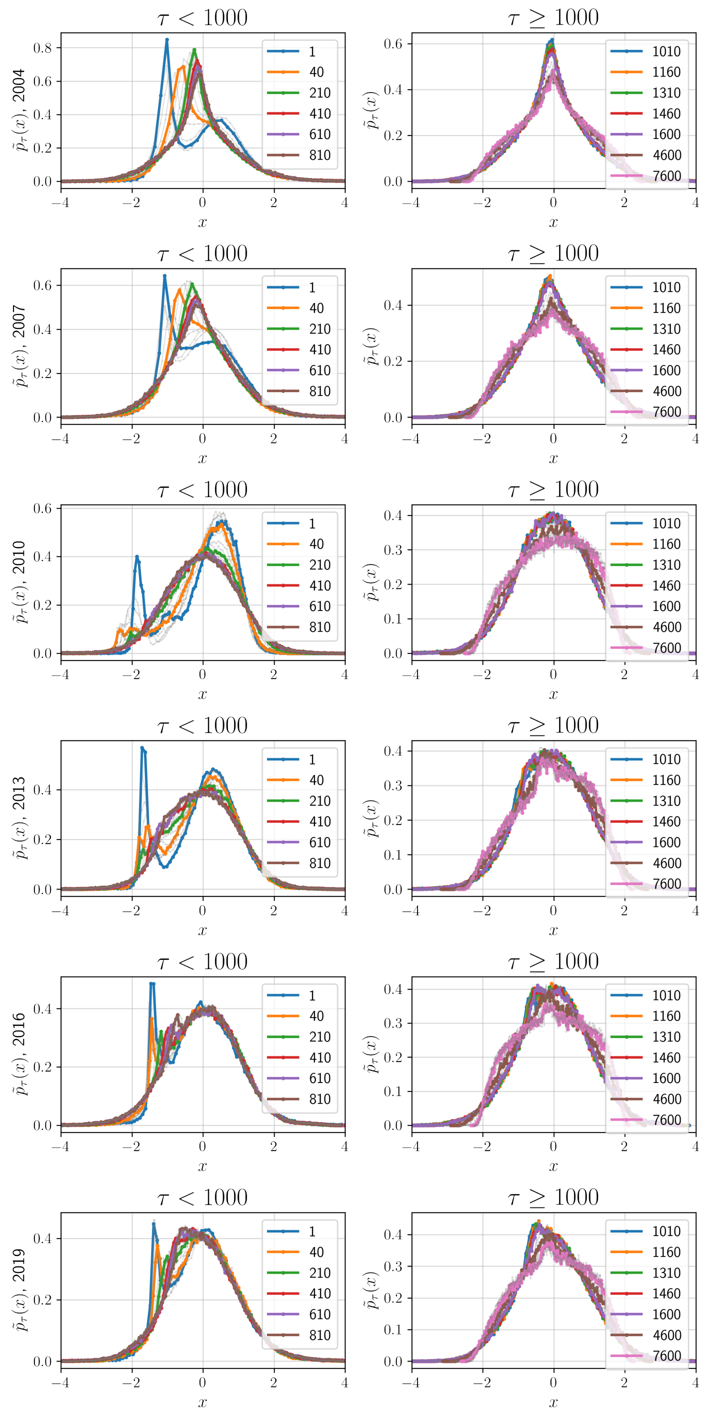

Fig 5. $T_{int} = 3$ **months - Qualitatively demonstrating the stability of the functional form for some** $\tau > \tau_0$: Standardized PDF $\tilde{p}_\tau(\cdot)$ visualized for 5 years - 1 year per row: (a) Left: $\tau = 1$ min to 1000 min and (b) Right: $\tau = 1000$ min to 10000 min. As τ exceeds 1000 min, the shape of $\tilde{p}_\tau(\cdot)$ takes a more stable form.

Deriving the modified vicsek model: By introducing the interaction terms in Eq. (9) into free particles described by the Langevin equation, we propose the following

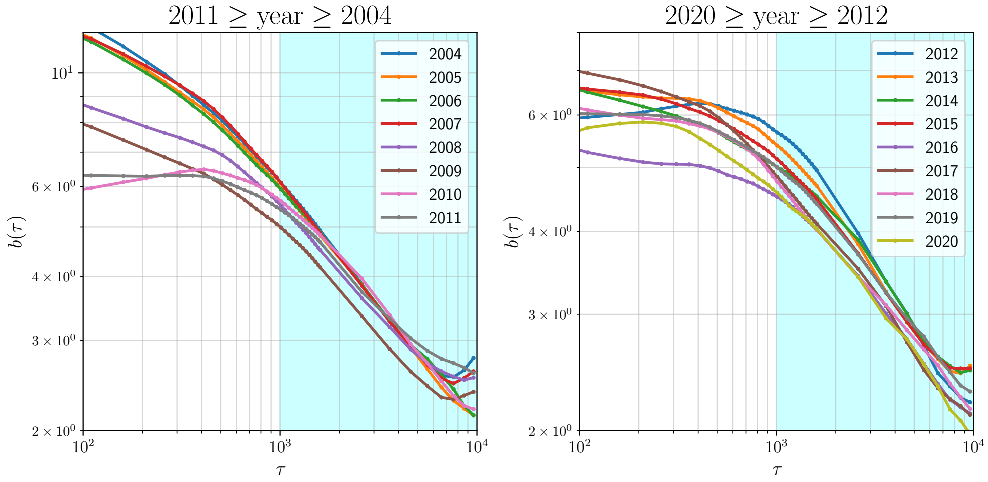

Fig 6. $T_{int} = 3$ **months - The power law (a) from 2004 to 2011; and (b) from 2012 to 2020** From $\tau = 1000$ min to 10000 min (the region highlighted by light cyan), observe the near-linear relationship between $\ln \tau$ and $\ln b$ similar to the case with $T_{int} = 1$ year.

model:

$$\dot{r}_i(t) = v_i(t), \tag{14a}$$

$$m_i \dot{v}_i(t) = -\alpha_i v_i(t) - \beta_i r_i(t) + \frac{\gamma_i}{N_{i,\delta}} \sum_{j:|r_i(t)-r_j(t)|<\delta} v_j(t) + \xi_i(t), \tag{14b}$$

where $N_{i,\delta}$ is the number of elements j that satisfy $|r_i(t) - r_j(t)| < \delta$ and

$$\xi_i(t) \coloneqq \frac{d}{dt} \Xi_i(t, \cdot). \tag{15}$$

Here we call Eq. (14) the modified Vicsek model. Note that $v_i(t)$ is included in $\sum_{j:|r_i(t)-r_j(t)|<\delta} v_j(t)$ if $\delta > 0$ and the summation is zero if $\delta = 0$. Here, for $t \geq t'$, $\Xi(t, t')$ obeys

$$\Xi_i(t, t') \sim \mathcal{N}(0, \eta_i^2 (t - t')). \tag{16}$$

Note that, in the case of the Langevin equation, we have $\eta_i^2 = 2\gamma k_{\mathrm{B}} T$.

For numerical simulations, we simplify Eq. (14). Unifying the two equations, Eq. (14), we obtain

$$m_i \ddot{r}_i(t) = -\alpha_i \dot{r}_i(t) - \beta_i r_i(t) + \frac{\gamma_i}{N_{i,\delta}} \sum_{j:|r_i(t)-r_j(t)|<\delta} \dot{r}_j(t) + \xi_i(t). \tag{17}$$

Taking the overdamped limit $m_i \to 0$, we get

$$\alpha_i \dot{r}_i(t) = -\beta_i r_i(t) + \frac{\gamma_i}{N_{i,\delta}} \sum_{j:|r_i(t)-r_j(t)|<\delta} \dot{r}_j(t) + \xi_i(t). \tag{18}$$

By discretizing Eq. (18), we have

$$\alpha_i r_i(t + \Delta t) = (\alpha_i - \beta_i \Delta t) r_i(t) + \frac{\gamma_i}{N_{i,\delta}} \sum_{j:|r_i(t)-r_j(t)|<\delta} (r_j(t + \Delta t) - r_j(t)) + \Xi_i(t + \Delta t, t). \tag{19}$$

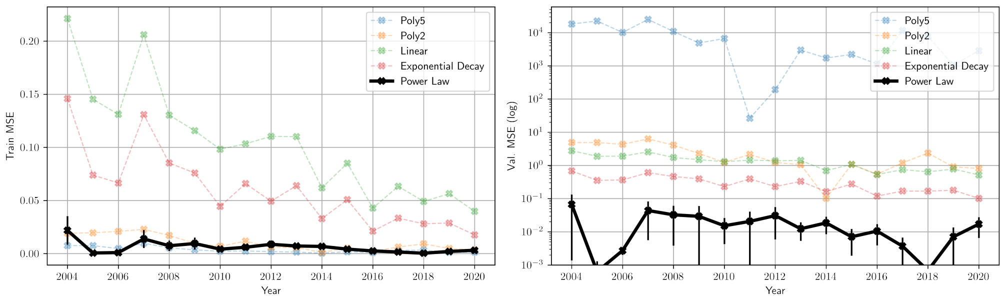

Fig 7. $T_{int} = 3$ **months - Model Architecture Search: Training and Validation MSE** The MSE (CI = σ) is reported as a measure-of-fit of every model for each year. Observe that the Power Law and the Stretched Exponential fits consistently reports lower validation MSE barring 2014. Error bars are computed across 4 folds of cross-validation. Polynomial models demonstrate clear signs of overfitting while the exponential model is only slightly worse in fit compared to the power law. These results are similar to the case with $T_{int} = 1$ year.

However, it is time-consuming to solve Eq. (19) since it involves a backward term. Assuming $r_j(t + \Delta t) - r_j(t) = r_j(t) - r_j(t - \Delta t)$, we have

$$\alpha_i r_i(t + \Delta t) = (\alpha_i - \beta_i \Delta t) r_i(t) + \frac{\gamma_i}{N_{i,\delta}} \sum_{j:|r_i(t) - r_j(t)| < \delta} (r_j(t) - r_j(t - \Delta t)) + \Xi_i(t + \Delta t, t). \tag{20}$$
